# Acute Lymphoblastic Leukemia in a Pediatric Patient With Turnpenny-Fry Syndrome

**DOI:** 10.7759/cureus.53099

**Published:** 2024-01-28

**Authors:** Inês Patrício Rodrigues, Beatriz Teixeira, Ana Miguel Capela, Marta Almeida, Cláudia Falcão Reis

**Affiliations:** 1 Pediatrics, Centro Hospitalar de Trás-os-Montes e Alto Douro, Vila Real, PRT; 2 Pediatrics, Centro Materno-Infantil do Norte, Centro Hospitalar e Universitário de Santo António, Porto, PRT; 3 Medical Genetics, Centro de Genética Médica Jacinto Magalhães, Centro Hospitalar Universitário de Santo António, Porto, PRT; 4 Pediatric Oncology, Instituto Português de Oncologia do Porto Francisco Gentil, Porto, PRT; 5 Unit for Multidisciplinary Research in Biomedicine, Abel Salazar Biomedical Sciences Institute, Porto University, Porto, PRT; 6 Life and Health Sciences Research Institute (ICVS), University of Minho, Campus de Gualtar, Braga, PRT; 7 ICVS/3B’s, PT Government Associated Laboratory, Braga/Guimarães, PRT

**Keywords:** developmental disabilities, pcgf2 gene, acute lymphoblastic leukemia, medical genetics, turnpenny-fry syndrome

## Abstract

Turnpenny-Fry Syndrome (TPFS) is a rare genetic disorder characterized by a severe developmental delay and a distinctive facial gestalt. It is caused by mutations in the Polycomb Group Ring Finger Protein 2 (PCGF2) gene, which is also known to play a role in numerous tumor types. Up to date, there have been no published case reports of patients with TPFS and concomitant malignancies. The present case describes the clinical evaluation and follow-up of a male infant with severe global developmental delay (GDD) and a distinctive phenotype. At 4 years of age, clinical exome sequencing confirmed the diagnosis of TPFS. Posteriorly, at 5 years of age, the patient was also diagnosed with T-cell acute lymphoblastic leukemia (ALL). Given the scarce literature regarding this syndrome, the authors expect that this case report will provide valuable information that could improve the follow-up of patients with TPFS. Furthermore, this case highlights the necessity for the appropriate diagnosis of developmental disorders, to ensure adequate care, surveillance of comorbidities and proper genetic counselling.

## Introduction

Turnpenny-Fry syndrome (TPFS) is a rare genetic disorder, characterized by a distinctive phenotype and severe developmental delay [1]. This syndrome was first described in 2018 by Turnpenny et al., who reported 13 patients, sharing similar facial features, global developmental delay (GDD), and heterozygous missense variants on the same residue of the Polycomb Group Ring Finger Protein 2 (PCGF2) gene [1]. Two of these patients had been previously identified in the “Deciphering Developmental Disorders Study”, published in 2015 [2].

To date, PCGF2 is the only known causative gene for TPFS [1]. It is expressed in various human tissues and is a component of a multiprotein complex called the polycomb repressive complex (PRC) 1, which is essential for regulating transcription in embryonic and adult stem cells [1]. PRCs function as epigenetic repressors of gene expression, that can act both as oncogenes and tumor suppressors [3]. PCGF2 plays a role in tumor suppression in breast cancer cells and was previously shown to negatively regulate granulocytic differentiation in human acute promyelocytic leukemia cells [4,5].

Following the first cases described by Turnpenny et al., only two additional patients with TPFS have been reported [6,7]. Thus, the complete understanding of this syndrome is limited by the small number of clinical reports. Furthermore, there have been no previous descriptions of patients with TPFS and malignant disorders, despite the implications of PCGF2 in oncogenetics [1,4,5]. With this case report we intend to raise awareness about this condition, while presenting, to our knowledge, the first patient with TPFS and a concomitant lymphoproliferative disease. The patient's guardians have given informed consent to document this report.

## Case presentation

The patient was a five-year-old male, the second child of a non-consanguineous couple. He was the product of a surveilled pregnancy. The mother was a healthy 36-year-old woman. First-trimester ultrasound revealed an increased nuchal translucency, with adequate fetal biometry. Chorionic villus sampling identified a normal karyotype (46, XY). Third-trimester ultrasound showed polyhydramnios, and the pregnancy was otherwise uneventful.

The patient was born at 40 weeks via induced vaginal delivery. His Apgar score was 6 and 7 at the first and fifth minute, respectively. He weighed 3,260g (38th percentile according to the Intergrowth-21st charts), had a length of 51cm (75th percentile) and an occipitofrontal circumference (OC) of 37cm (98th percentile). On examination he was hypotonic and had distinctive features, with a flat facial profile, narrow palpebral fissures, and dysplastic ears. After initial feeding difficulties, he acquired progressive autonomy. Follow-up was marked by severe neurodevelopmental delay. He acquired head control at six months of age, was able to sit at 12 months, crawled at 19 months and walked unsupported at four years. Presently, he remains non-verbal, has a generally calm temper but is easily frustrated, particularly by auditory stimuli.

**Figure 1:**
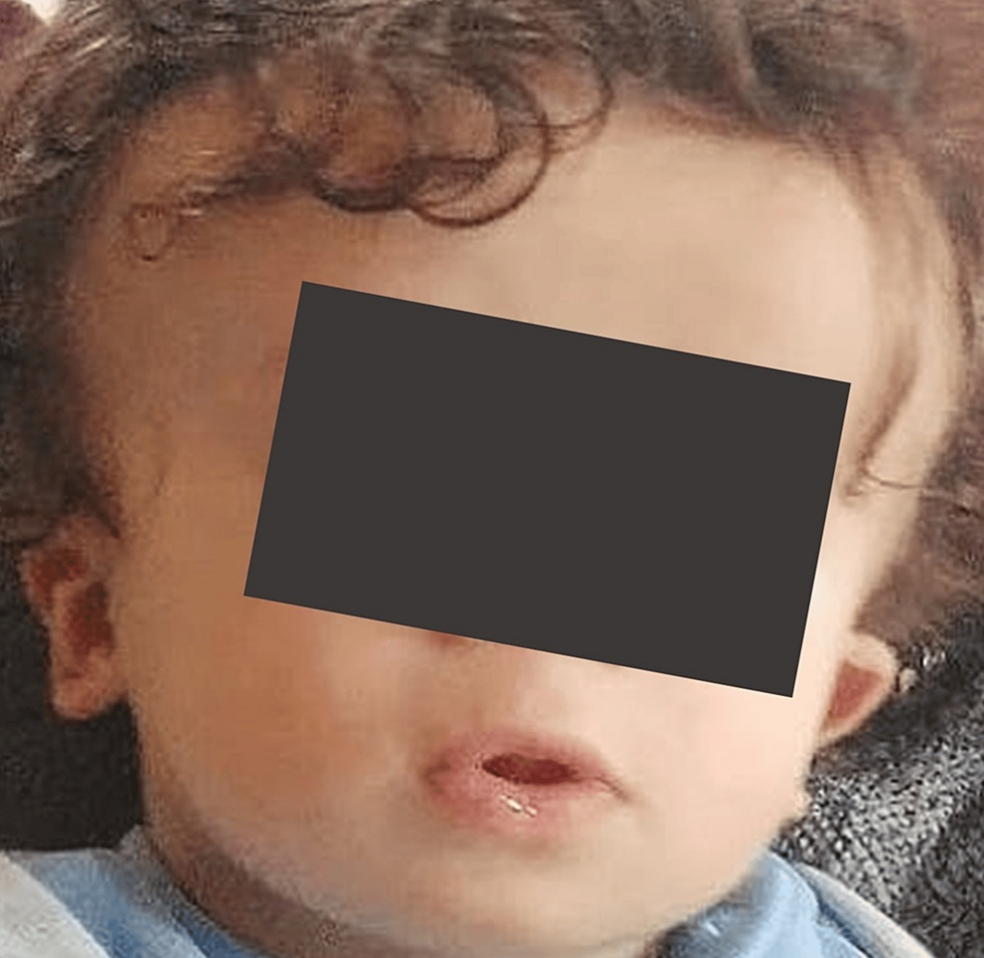
Patient at six months of age. The patient's appearance was characterized by macrocephaly, frontal bossing, periorbital fullness, narrow palpebral fissures and low-set, dysplastic ears.

**Figure 2:**
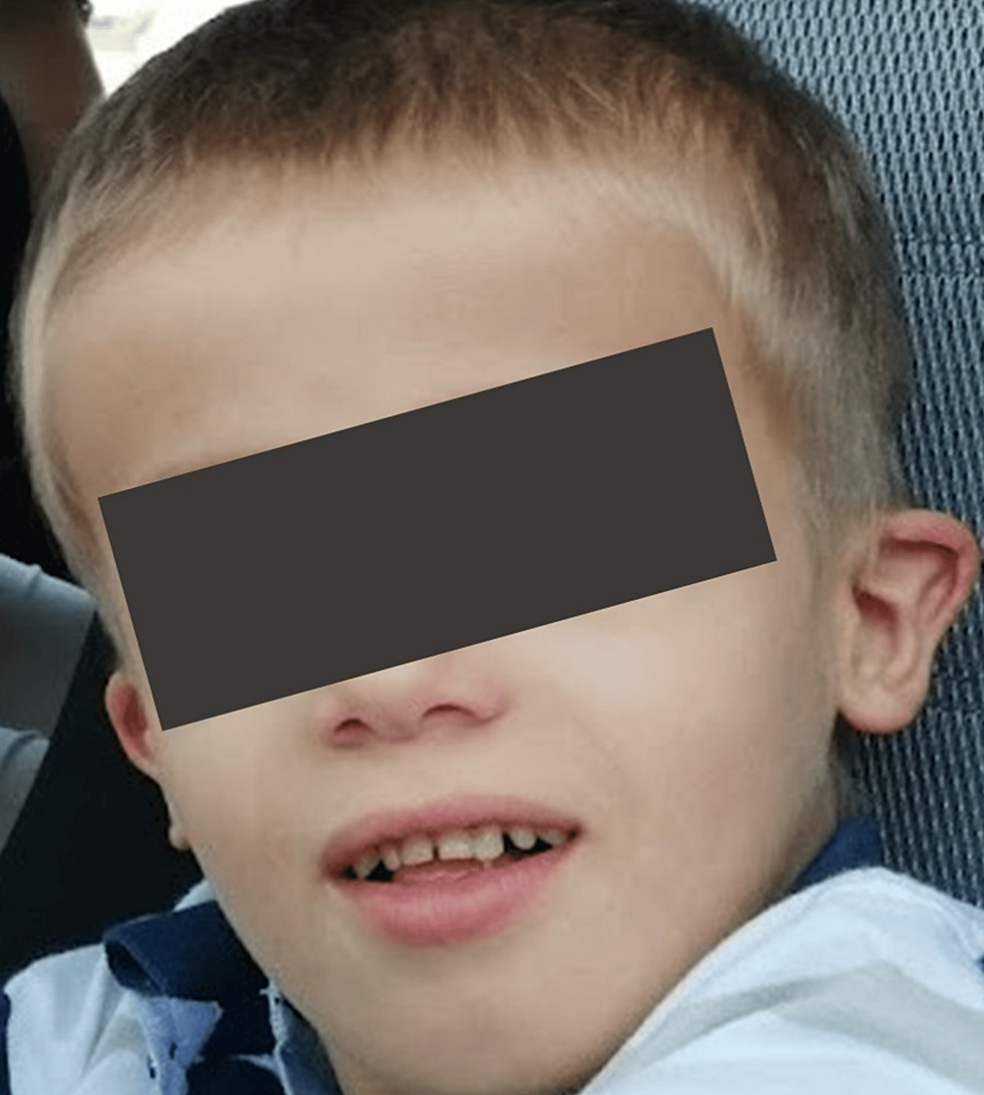
Patient at five years of age. To be noted the open mouth posture and low-set, dysplastic, ears.

Growth was characterized by an adequate weight and height evolution and mild macrocephaly. His facial features became increasingly apparent, with marked frontal bossing and mid-face hypoplasia, giving a triangular appearance to his lower face (Figure 1, 2). He developed a high-arched palate, with dental crowing and a supernumerary maxillary canine. He had an open mouth resting posture, with frequent drooling, and suffered from constipation.

Brain MRI at 10 months showed mild lateral ventriculomegaly. Metabolic studies, karyotype and microarray were normal. X-fragile syndrome testing was negative. At 4 years, clinical exome sequencing (mendeliome) identified a heterozygous variant in the PCGF2 gene, (NM_007144.2), c.194C>T, p.(Pro65Leu), previously reported as pathogenic, and which was presumed to have occurred *de novo* after negative parental testing. This finding established the diagnosis of TPFS.

The patient was submitted to further evaluations. Echocardiogram was normal. Ophthalmic examination revealed mild tortuosity of the retinal vasculature. Skeletal survey identified bilateral dysmorphic calcaneus and astragalus bones. Further description is presented in Table 1.

**Table 1:** Phenotypical characteristics of the presented patient and comparison with prior reported cases. GDD: Global developmental delay; N/K: Information not known or not mentioned in prior reports; (+): Positive finding; (-): Characteristic not present *For some characteristics, data was not available for all patients.

Author, year	Present Case	Ercoskun et al. (2021) [6]	Qi et al. (2022) [7]	Turnpenny et al. (2018) [1]
Number of cases	1	1	1	13^*^
Antenatal				
Increased nuchal translucency	+	N/K	N/K	1/13
Polyhydramnios	+	N/K	N/K	5/13
Intrauterine growth restriction	-	N/K	N/K	3/13
Birth				
Term	+	+	+	13/13
Low-birth weight (< 10thpercentile)	+	+	+	8/13
Craniofacial features				
Macrocephaly	+	-	-	2/13
Microcephaly	-	-	-	4/13
Frontal bossing	+	+	+	13/13
Sparse hair	-	+	+	12/12
Periorbital fullness	+	+	+	12/12
Narrow palpebral fissures	+	+	N/K	11/12
Prominent nasal tip	+	+	N/K	10/13
Small mouth aperture	+	-	+	7/12
High palate	+	N/K	N/K	6/6
Abnormal dentition	+	N/K	N/K	9/13
Mandibular prognathia	+	-	N/K	7/13
Malar hypoplasia	+	+	+	12/12
Dysplastic ears	+	+	+	12/12
Skeletal				
Vertebral hypoplasia	-	N/K	N/K	4/13
Kyphosis/scoliosis	-	N/K	N/K	6/13
Pectus deformities	-	N/K	N/K	3/13
Feet deformities	+	N/K	N/K	6/10
Cardiac				
Cardiac defects	-	+	N/K	11/13
Genitourinary defects				
Undescended testicles/cryptorchidism	-	N/K	N/K	2/13
Gastroenteric				
Feeding difficulties in infancy	+	N/K	N/K	9/13
Gastroesophageal reflux	-	N/K	N/K	6/13
Constipation	+	+	N/K	8/12
Drooling	+	N/K	N/K	3/13
Diaphragmatic hernia	+	-	N/K	2/13
Otolaryngologic				
Hearing loss	-	+	N/K	7/13
Ophthalmologic				
Strabismus	+	+	N/K	N/K
Transient corneal opacities	-	N/K	N/K	2/13
Tortuous retinal vasculature	+	N/K	N/K	1/13
CNS				
Brain MRI alterations (ventricular dilation)	+	+	+	2/13
Brain MRI alterations (abnormal white matter)	-	+	+	9/13
Brain MRI alterations (polymicrogyria)	-	-	-	4/13
Intellectual disability/GDD (mild to severe)	+	+	+	13/13
Delayed or absent speech	+	+	+	13/13
Hypotonia	+	+	N/K	9/13

At five years, the patient presented to the emergency-department with a one-week history of non-productive cough, anorexia, and low-grade fever. On examination he was pale, had extensive ecchymosis and multiple subcentimetric lymphadenopathies in the cervical, axillar, and inguinal regions. He also presented with significant hepatosplenomegaly.

**Figure 3:**
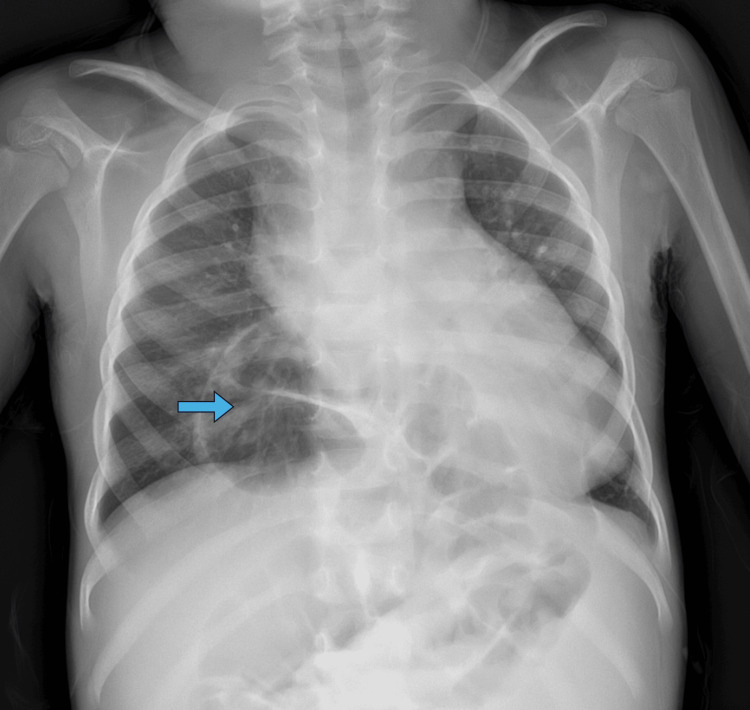
Chest radiography demonstrating herniation of the colon into the thorax (blue arrow).

**Figure 4:**
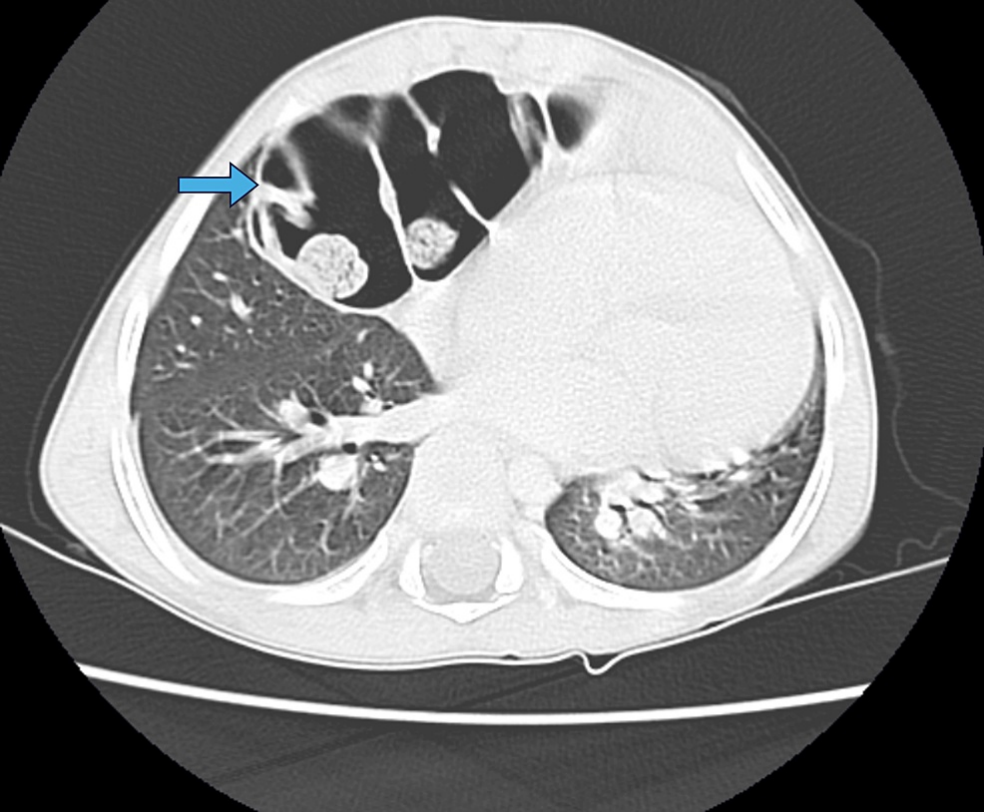
Axial section of the chest CT scan showing herniation of the colon into the thorax (blue arrow).

Laboratory results disclosed marked leukocytosis, anemia, and thrombocytopenia, as described in Table 2. Chest radiography identified a previously undiagnosed diaphragmatic hernia (Figure 3). CT confirmed the presence of a voluminous anteromedial diaphragmatic hernia, with no acute complications (Figure 4). Lung expansion was normal. There were generalized lymphadenopathies in the mediastinum, cervical and axillary regions, as well as in the abdominal groups, and para-aortic chains.

**Table 2:** Pertinent laboratory results. WBC: White blood cell count; ALT: Alanine transaminase; AST: Aspartate transaminase *Values adjusted to the patient’s age group.

Laboratory Parameters	Results	Reference Range*
Hemoglobin	5.6 g/dL	11.7–14.1 g/dL
Hematocrit	18.8 %	35–44 %
WBC	80,900 /µL	5,000–15,500 /µL
Lymphocyte count	22,500 /µL	2,000–8,000 /µL
Neutrophil count	500 /µL	1,500–7,500 /µL
Peripheral blood blasts	92.7%	0 %
Platelets	16000 /µL	150,000–450,000 /µL
Erythrocyte sedimentation rate	120 mm/hr	0–10 mm/hr
Prothrombin time	12.5 seconds	12.5–15.2 seconds
Partial thromboplastin time	24 seconds	25–35 seconds
International normalized ratio	1.1	0.9–1.3
Sodium	139 mmol/L	135–145 mmol/L
Potassium	4.5 mmol/L	3.9–5.3 mmol/L
Chloride	106 mmol/L	98–105 mmol/L
Total serum calcium	2.22 mmol/L	2.2–2.7 mmol/L
Magnesium	1.01 mmol/L	0.7–1.5 mmol/L
Creatinine	44.21 µmol/L	25–42 µmol/L
Urea	5.9 mmol/L	2.5–6.0 mmol/L
Uric Acid	410 µmol/L	34–103 µmol/L
ALT	8 IU/L	< 34 IU/L
AST	26 IU/L	14–49 IU/L
Lactate dehydrogenase	1717 IU/L	150–300 IU/L
Total protein	73 g/L	60–80 g/L
Albumin	38 g/L	40–50 g/L
C-reactive protein	28.2 mg/L	<20 mg/L

The patient was transferred to the Pediatric Department of a third-level oncology hospital for further investigation. On flow cytometry of peripheral blood, blasts accounted for 94% of the total leucocyte count. Based on morphologic and immunophenotyping results, a diagnosis of T-cell acute lymphoblastic leukemia (ALL) was rendered.

All subsequent genomic studies were performed on peripheral blood. Conventional chromosome evaluation identified a complex 46, XY karyotype. Fluorescent *in situ* hybridization (FISH) study was concordant with karyotyping, and identified a deletion of region 5q32~33 (genes CSF1R and PDGFRB) in 81% of the analyzed cells, and a rearrangement involving the TRAD locus (14q11) in 24%, with no other genetic alterations noted.

Cerebral spinal fluid was negative for malignant cells and there was no identifiable testicular disease. According to the Alltogether-1 Clinical Trial Protocol, the patient was classified as being intermediate-high risk and initiated systemic chemotherapy accordingly. Currently, he is 18-months post diagnosis, under maintenance therapy, with favorable response.

## Discussion

Recent scientific progress has made it possible to identify the genetic causes underlying many cases of neurodevelopmental delay, therefore improving the management of these patients. TPFS has been recently described as a genetic disorder associated with severe developmental delay, with only 15 reports published to date. The present case is, as far as the authors are aware, the 16th patient reported with TPFS.

The phenotypical description of the presented patient, as well as the respective comparison with previously reported cases, is provided in Table 1. All patients share the same characteristic facial gestalt, which is an important clue for the diagnosis [1,6,7]. Prenatally, our patient presented with an increased nuchal translucency and polyhydramnios, having both alterations been previously mentioned in other cases [1]. Unlike most prior reports, our patient had an adequate birth weight. The incidental finding of a diaphragmatic hernia, a malformation associated with significant morbidity that had also been reported in two other patients, is also noteworthy [1]. Upon the diagnosis of TPFS, all major comorbidities known to be associated with this syndrome should actively be investigated and surveilled. 

In 2018, Turnpenny et al. highlighted that, even though PCGF2 was implicated in tumor suppression, none of the reported patients had been diagnosed with malignancy [1]. To our knowledge, this is a unique case of a patient with childhood T-ALL and TPFS. It is known that ALL is the most common childhood malignancy, accounting for approximately 25% of all pediatric cancers, with a peak incidence between two to five years, the same age group as the presented patient [8]. In the last two decades, there has been a continuous improvement of the 10-year survival rate for pediatric ALL, which is now around 90% [8]. Early diagnosis and prompt treatment have significantly contributed to this improvement. The authors cannot infer any causal association between TPFS and ALL. However, given the role of PCGF2 in tumor suppression, as well as the scarce literature regarding TPFS, the overlapping of both conditions is worth noting. To infer any further conclusions, it is fundamental that clinicians are aware of this syndrome and report future cases, specially if associated with malignancy. 

## Conclusions

Given the limited data concerning patients with TPFS, and the fact that its association with leukemia has never been previously reported, the authors expect that this report will provide valuable information that could improve the follow-up, as well as the anticipation of any possible complications. Furthermore, this case underlines the importance of the appropriate etiological investigation of developmental disorders to ensure proper care, surveillance of comorbidities, and genetic counselling.
